# Comparison of serum levels of vitamin D and vitamin D-binding protein in normal, osteopenic and osteoporotic postmenopausal women

**DOI:** 10.12669/pjms.35.2.714

**Published:** 2019

**Authors:** Rafat Murad, Tabassum Mahboob, Rehana Rehman, Rozeena Baig

**Affiliations:** 1 *Prof. Rafat Murad, MBBS, M.Phil. Department of Biochemistry, BMSI, JPMC, Karachi, Pakistan*; 2 *Prof. Tabassum Mahboob, PhD. Department of Biochemistry, University of Karachi, Karachi Pakistan*; 3 *Dr. Rehana Rehman, MBBS, M.Phil., PhD. Associate Professor, Department of Biological & Biomedical Sciences, Aga Khan University, Karachi, Pakistan*; 4 *Rozeena Baig, MSc. Department of Biological & Biomedical Sciences, Aga Khan University, Karachi, Pakistan*

**Keywords:** Vitamin D, Vitamin D binding protein, Bone mineral density, Post menopausal females

## Abstract

**Objective:**

To compare the serum levels of vitamin D, vitamin D binding protein (VDBP) calcium and phosphate in normal, osteopenic and osteoporotic postmenopausal women categorized on the basis of bone mineral density (BMD) scores.

**Methods:**

A cross sectional study carried out from May 2017 to August 2018. BMD measured by Dual energy X-ray Absorptiometry categorized women (aged 20- 70 years) into normal (n=37) (T score ≥ -1.0) osteopenic (n=25) (-2.5< T score, < -1) and osteoporotic (n= 26) (T score < -2.5) according to WHO classification. Serum concentrations of vitamin D, VDBP, calcium, phosphate analyzed by enzyme linked immunosorbent assay were compared by Analysis of Variance

**Results:**

In normal females higher levels of vitamin D and VDBP were observed [15.82 (8 - 69.18), 469.9 (269.57 - 875.55)] vs. osteopenic [(7.45 (4.66 - 15.1), 296.05 (232.58 - 420.23)] and osteoporotic women [(7.25 (3.97 - 17.49), 272.94 (202.23 - 351.24)]; [median interquartile range]; p value < 0.0001.

**Conclusion:**

Vitamin D and VDBP are linked with bone health and estimation of VDBP appears to be a valuable tool for the assessment of increased bone loss and possible risks of bone fractures especially in postmenopausal women.

## INTRODUCTION

Vitamin D deficiency is common in general population.^1^ About one billion people suffer from vitamin D deficiency around the globe.^2^ Adequate vitamin D is essential for maintaining skeletal growth owing to its central role in calcium and phosphate metabolism.^3^ Vitamin D ensures the mineralization of the organic matrix of bone, and also mediates the release of calcium and phosphate from bone to achieve mineral homeostasis.^4^ Calcium and phosphate not only helps in achieving peak bone before the onset of menopause and age related decline in bone formation but also provide protection against bone loss and fractures.^5^

Low levels of vitamin D have been shown to have adverse effects on calcium metabolism, osteoblastic activity, matrix ossification, bone mineral density (BMD). Deficiency of vitamin D leads to the development of osteomalacia (incomplete mineralization of osteoid) in adults and rickets in children. Chronic insufficiency of vitamin D leads to secondary hyperparathyroidism with increased bone turnover and decreased BMD. The progressive bone loss exacerbates both osteopenia and osteoporosis and increased risk of fragility fractures especially in elderly individuals.^6^

Concentration of vitamin D within the body is affected by various factors including age, body weight, ethnicity, genetic polymorphisms and vitamin D–binding protein (VDBP), the primary vitamin D carrier protein.^7^

Majority of vitamin D in serum is bound to VDBP (80-90%), and also bound to albumin and chylomicrons (10 – 15%) at lower levels and affinity, very small fraction is free (<0.1%) and is biologically active. Vitamin D bound to VDBP, facilitates the access of vitamin D to various tissues and cells and regulates the total amount of vitamin D available for the organism. It protects vitamin D metabolites from hydroxylase-mediated catabolism and therefore act as vitamin D reservoir.^8^ The hepatic synthesis of VDBP is stimulated by estrogen as seen in various studies that showed increase in serum concentration of VDBP in pregnant females and in women taking exogenous estrogen.^9^

Menopause either involutional or estrogen related is associated with increased bone turnover and risk of osteoporotic fracture owing to multiple factors including low levels of total circulating vitamin D and VDBP.^10^ The current study was designed to compare the serum levels of vitamin D, VDBP, calcium, phosphate along with biophysical parameters in normal, postmenopausal osteopenic and osteoporotic women and also to examine the relationship between vitamin D, VD, BP and BMD in all three groups.

## METHODS

This cross-sectional study comprised of 88 healthy women (aged 20-70 years). Study was conducted in the Department of Biological & Biomedical Sciences Aga Khan University from May 2017 - August 2018 after approval from Ethical Committee of Aga Khan University (4146-BBS-ERC-16). Combined prevalence of osteoporosis and osteopenia is 82%.^11^We calculated 57 sample size for osteopenic and osteoporotic group using infinite formula for sample size calculation for osteoporosis and osteopenic groups with 95% confidence level (1.96), prevalence 82% and margin of error 10% in the following formula;

n=(Z^2 pq)/e^2

n=((1.96)^2 (0.82)(0.18))/ (0.1) ^2

n=57

We collected data of 57 subjects, but due to drop out of 6 subjects, we compared 51 postmenopausal women with minimum significant normal (37) subjects

Where: z = standard normal deviation set at 95% confidence level

p = percentage picking a choice or response

c = confidence interval

The subjects were recruited from the community, motivated to come to AKU where their free DEXA scans were done and blood samples were acquired after taking written informed consent from each participant. Weight was recorded digitally to the nearest 0.1kg while height was recorded to the nearest 0.1cm using seca 217 stadiometer. Body mass index (BMI) was calculated as the ratio of weight (kg) and height (m^2^).

**Fig.1:**
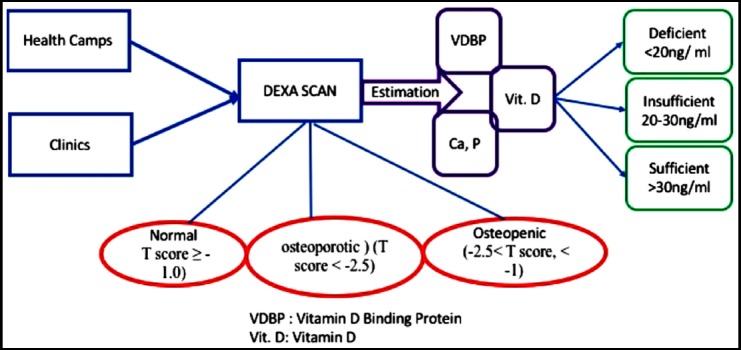
Patient selection flow chart.

Subjects were recruited by organizing health camps in different districts of Karachi through convenient sampling (Fig.1). Participants with history of premature menopause, vertebral fractures or suffering from systemic illness like hyperthyroidism, hyperparathyroidism, renal failure or taking any medications known to affect bone metabolism like bisphosphonates, calcium, vitamin D supplements, calcitonin, hormone replacement therapy and anabolic steroids were excluded from the study. Women were further categorized on the basis of BMD measurements according to WHO classification into, normal (T score ≥ -1.0) (n=37) osteopenic (n=25) (-2.5< T score, < -1) and osteoporotic (n= 26) (T score < -2.5).^12^ BMD in (gm/cm^2^) was measured by DEXA scan by Hologic discovery A (S/N 80855). Sites of measurement were hip, femoral neck and spine (L1-L4).

Serum vitamin D, VDBP, calcium and Phosphate were measured by solid phase enzyme amplified sensitivity immunoassay method (commercial kit DIA source immunoassay, Belgium) kitCat≠95503 for vitamin D, kit Cat ≠ 97145 for VDBP). Calcium and phosphate were measured by kit Cat≠ SP 1001061, and kit Cat ≠ SP1001150 respectively. Cut off values for deficient, insufficient and sufficient vitamin D levels were taken to be <20ng/ ml, 20-30ng/ml and >30ng/ml respectively.^13^ Statistical analyses were done using Statistical Package for Social Sciences SPSS (version 21). Baseline characteristics of the participants were analyzed by descriptive statistics (mean± SD). Statistical comparison was performed using ANOVA, post hoc Tukey HSD test was then applied. All p-values < 0.05 were taken as significant.

## RESULTS

Total 88 healthy women were included in this study, out of which 37 were normal, 25 were osteopenic and 26 were osteoporotic women as per estimation of BMD. In normal group (n=37) premenopausal women were (n=30, 11.1%), and postmenopausal women were (n=7, 2.5%), whereas osteopenic (n=25) and osteoporotic group (n=26) comprised only of postmenopausal women.

The characteristics of study participants are shown in Table-I. Serum levels of vitamin D and VDBP were significantly different across groups and were on higher side in normal compared to osteopenic and osteoporotic group (p value < 0.0001).

**Table-I:** Comparison of baseline characteristics of the participants.

	Normal (37)	Osteopenic (25)	Osteoporotic (26)	P value
Age (years)	36.43 ±13.71	52.48 ±10.324	58.577±5.021**^^	<0.001
Height (centimeters)	157.21 ±5.678	155.256±4.792	154±5.546	0.06
Weight	62.8 ± 12.886	67.12±11.641	60.904±9.846	0.15
BMI kg/m^2^	25.319 ±5.463	28.132±5.073	26.262±4.943	0.11
Duration of Menopause (years)		8.286±3.717	7.885±3.953	0.16
Vitamin D (ng/ml) Median (Interquartile Range)	15.82 (8 - 69.18)	7.45 (4.66 - 15.1)	7.25 (3.97 - 17.49) ^	0.06
VDBP (ng/ml) Median (Interquartile Range)	469.9 (269.57 - 875.55)	296.05 (232.58 - 420.23)	272.94 (202.23 - 351.24*^	0.01
Calcium mg/dl	7.643±1.37	7.3±1.1216	7.86±1.559	0.35
Phosphate mg/dl	4.385±1.084	4.572± 1.033	4.457±1.11	0.80
Dexa Hip score	-0.543±0.803	-0.728±0.648	-1.95± 0.62	<0.001
Dexa score spine	0.797±0.65	-1.556±1.023	-2.95±1.047	<0.001
Dexa Hip femoral neck	0.927±0.705	-1.476±0.63	-2.527±0.556	<0.001

Values are expressed in mean±Standard deviation or Median (Interquartile range) Analysis of variance was used to compare means between groups.

* p value<0.05 is significant in osteoporotic group as compared to osteopenic

** p value<0.001 is significant osteoporotic group as compared to osteopenic

^ p value<0.05 is significant in osteoporotic group as compared to normal

^^ p value<0.001 is significant in osteoporotic group as compared to normal.

**Fig.2:**
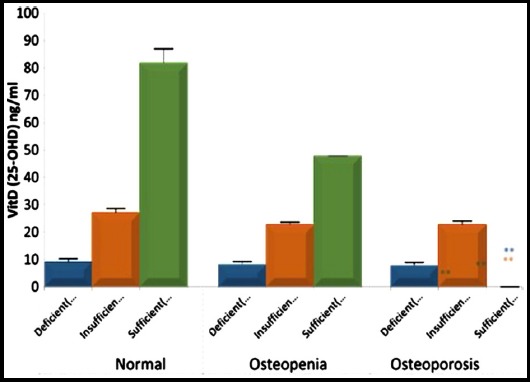
Vitamin D levels in normal, osteopenic and osteoporotic females on the basis of cut off values deficient, insufficient and sufficient vitamin D levels were taken to be<20ng/ ml, 20-30ng/ml and >30ng/ml.

The Fig.2 describes the distribution of normal, osteopenic and osteoporotic groups on the basis of cut off values of vitamin D (deficiency< 20ng/ml, insufficiency 20-30ng/ml, sufficiency > 30ng/ml.^13^ It shows that in normal group (n=37) the number of females deficient, insufficient and sufficient for vitamin D levels were 35%, 22% and 43% respectively. Vitamin D levels in normal women differed significantly from osteopenic group 56% deficient, 28% insufficient and 16% sufficient. In osteoporotic group 62% females were deficient, 23% were insufficient and 15% had sufficient Vitamin D levels respectively (p value <0.0001).

**Fig.3:**
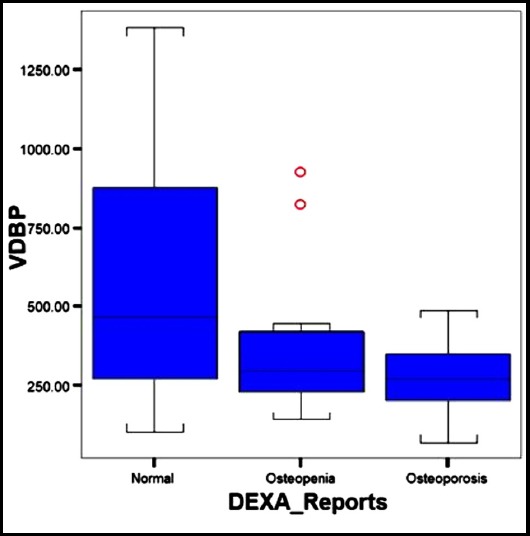
Comparison of Vitamin D Binding Proteins in the Study Groups.

Fig.3 describes the representation of median values of VDBP in all study groups. In normal group, serum VDBP levels were higher (594.583±397.601 ng/ml) compared to osteopenic (348.524±183.501 ng/ml) and osteoporotic group (278.934±109.582ng/ml) (p value < 0.0001).

## DISCUSSION

Hypovitaminosis D is common worldwide and affects children and elderly people predominantly postmenopausal women.^14^,^15^ Our study shows that serum levels of vitamin D and VDBP were lower in postmenopausal osteopenic and osteoporotic women compared to the normal group. Similar findings can be compared by another study which explained lower levels of estrogen as a contributing factor for low vitamin D concentration in postmenopausal women.^16^ Present study also showed higher mean age in osteoporotic group compared to the normal group which also suggests an age-related phenomenon responsible for decreased vitamin D levels in these women. Impaired function of the enzyme 1 α hydroxylase required for the formation of active vitamin D (1, 25 dihydroxycholicalciferol) and increased metabolic clearance of vitamin D metabolites is responsible for a decline in vitamin D levels seen in elderly postmenopausal osteoporotic women.^17^ Results of this study also showed that BMD measured at hip, spine and femoral neck was also significantly lower in postmenopausal osteopenic and osteoporotic women having lower serum vitamin D levels compared to the normal group. Similar finding are reported by Nakamura et al showing decline in BMD at femoral neck in subjects having low vitamin D levels compared to those having adequate levels of vitamin D.^18^

Serum calcium and phosphate were low in all three study groups, as shown in our results; however no significant difference was seen among three groups. This may be related to either low intake or impaired absorption of minerals seen in vitamin D deficiency or inadequacy.^19^

Among postmenopausal osteoporotic women the prevalence of vitamin D deficiency was 62% using a cut off value <20ng/ml and insufficiency was 23%, using a cut-off of 20-30ng/ml. Previous literature has reported increased serum levels of parathyroid hormone in subjects having low serum vitamin D levels < 20ng/ml causing decrease in calcium absorption and serum calcium concentration, which is maintained as the expense of increased parathyroid secretion, referred to as secondary hyperparathyroidism.^20^ Several observational studies have reported the association of serum vitamin D levels with osteoporotic fractures. A cohort study conducted by *Tanaka et al* comprising of 1470 postmenopausal women revealed that those with serum vitamin D levels < 25ng/ml showed a significant higher risk of long bone fracture.^21^ Another study comprising of 1262 postmenopausal women have reported higher incidence of 5 year total fracture risk among postmenopausal women having basal serum vitamin D levels <10 ng/ml followed by those whose vitamin D levels were between 10-20ng/ml and 20-30ng/ml and was lowest in those having > 30ng/ml.^22^

Various factors are responsible for maintaining vitamin D status including VDBP which is responsible for maintaining the availability and regulation of serum vitamin D levels. This study results have shown that VDBP concentration was lower in osteoporotic women compared to normal group of women. Others have also shown decreased levels of VDBP in females than in males, which could be due to differences in estrogen levels.^23^ Increased serum VDBP levels have also been reported in women receiving hormone replacement therapy, oral contraceptives and in pregnancy.^24^ Similarly concomitant decreased concentration of VDBP and vitamin D in postmenopausal osteoporotic women as reported in our study, might be due to physiological adaptation to maintain free vitamin D levels or due to a compensatory mechanism of reduced hepatic synthesis of VDBP as result of decreased vitamin D in postmenopausal osteoporotic women.^25^

Our study also highlighted increased serum levels of VDBP in normal premenopausal women compared to postmenopausal osteoporotic women. This can be due to the deficiency of estrogen hormone in postmenopausal women, which is required in higher concentration to raise serum levels of VDBP as identified in previous estrogen administration studies and during pregnancy.^26^

This study results have identified the role of vitamin D and VDBP for evaluating the risk of osteoporosis and osteoporotic related fractures in postmenopausal women, but due to limited number of patients, we could not draw conclusions from the findings. Further studies with large sample size are needed to assess the role of vitamin D and VDBP in the prediction of loss of BMD and risk of fragility fractures especially in postmenopausal osteoporotic women. We have not related our results with exposure to sun, latitude, season, time of day, and atmospheric components as all subjects were permanent residents of Karachi. Also initial screening at camps ruled out subjects positive for certain other factors like obesity, chronic illnesses and smoking etc.

## CONCLUSION

Serum levels of vitamin D and VDBP were lower in osteopenic and osteoporotic post-menopausal women as compared to the normal females who reflected normal bone architecture as evidenced by normal BMD. Vitamin D and VDBP are thus important for incremental increase in BMD, furthermore recommend that VDBP may be used as a valuable tool to identify the risk of osteoporotic fractures associated with decline in BMD especially in postmenopausal women.

### Authors’ Contribution

**RR:** Conceived the idea.

**RM and TM:** Designed & executed the study, did statistical analysis and took part in the writing of the manuscript.

**RB:** Collected the data, helped in statistical analysis and manuscript writing.

All authors reviewed for final approval of the manuscript.
